# A Review of Management of Inflammation in the HIV Population

**DOI:** 10.1155/2016/3420638

**Published:** 2016-09-27

**Authors:** Jihad Slim, Christopher F. Saling

**Affiliations:** ^1^Saint Michael's Medical Center, Newark, NJ 07102-2094, USA; ^2^Saint Joseph's Regional Medical Center, Paterson, NJ 07503, USA; ^3^New York Medical College, Valhalla, NY 10595, USA; ^4^Internal Medicine, Saint Michael's Medical Center, Newark, NJ 07102, USA

## Abstract

Advancements in antiretroviral therapy have drastically increased the life expectancy for those infected with HIV. Today, a new subgroup of older patients with long-term controlled HIV exists, and its populace is continuously mounting. Therefore, it is essential to understand the enduring effects of chronic suppressed HIV infection in order to further improve HIV management in these patients. This paper will examine the role of HIV in chronic inflammation and immune dysfunction, the dynamic interaction that exists between comorbidity and HIV, and the potential consequences of long-term antiretroviral therapy in an effort to provide the best management options for the virally suppressed HIV patient.

## 1. Introduction

Before the advent of effective cART, HIV infection was usually a death sentence. The virus would hijack the immune system unchecked, often leading to full-blown AIDS and acute opportunistic infection. According to the CDC, there were a total of 774,467 diagnosed cases of AIDS and 448,060 AIDS-related deaths in the USA from 1981 to 2000 [1]. Over the past two decades, cART has dramatically reduced the incidence of morbidity and mortality related to both HIV and opportunistic infections. These advancements have enabled most persons living with HIV (PLWH) to achieve an undetectable viral load (VL) within 12 weeks of the initiation of treatment. This has led to the emergence of a new and growing population of aging HIV-positive patients. The effects of chronic suppressed HIV infection in this group are only beginning to become understood, and methods to combat these effects are poorly studied.

Although the average life expectancy for PLWH has increased significantly, it is still slightly less than that of the general population. The highest estimated life expectancy for a newly diagnosed HIV-positive 20-year-old patient on cART in the USA or Canada is just above 70 years [2]. Reasons for this gap have become a new focus of HIV research. One theory is that even with undetectable VL and adequate CD4^+^ count there exists a state of persistent inflammation. Also, traditional comorbidities may become worsened by chronic HIV infection. Furthermore, long-term use of cART may lead to adverse effects that could further augment the severity of these comorbidities. This paper will focus on each one of these concepts in order to provide possible treatment measures to thwart their harmful effects on patients with suppressed HIV infection.

## 2. Materials and Methods

A computer-based search using Pubmed and Embase was conducted to review the literature regarding antiretroviral interventions to decrease chronic inflammation in patients on combination antiretroviral therapy (cART).

## 3. Results and Discussion

### 3.1. Inflammation and Dysregulation of the Immune System

Inflammatory markers appear to directly correlate with morbidity and mortality in patients infected with HIV. Since this inflammatory process, which is a correlate of T-cell activation, is much more pronounced when the virus is not suppressed, the discussion will be limited towards patients receiving effective treatment for HIV [3]. Although successful cART does not suppress all inflammatory mechanisms associated with HIV, it has been shown to decrease some immune activation markers to the level of HIV-uninfected individuals, particularly monocyte-macrophage activation [4]. However, in clinical practice HIV virus load (VL) is measured intermittently and there are different cut-offs for detection. Thus, it is conceivable that low-grade or intermittent viremia is actually occurring in patients that are classified as undetectable. This phenomenon could play a role in persistent inflammation.

It is also relatively well established that a chronic inflammatory state in patients receiving appropriate cART is primarily related to the extent of damaged gut-associated lymphoid tissue and its subsequent microbial translocation [5, 6]. This process involves commensal microbes from the gastrointestinal tract entering portal and systemic circulation [7]. Acute HIV infection is marked by an intense surge of cytokines such as interferon-*α*, interferon-*γ*, tumor necrosis factor, and IL-6 which leads to immune activation and severe inflammatory reaction [8]. This causes a profound depletion of CD4^+^ cells from the gut, which only partially improves with effective cART [9, 10]. This explains the reasoning behind intestinal microbial translocation and subsequent immune stimulation [11]. One method for studying microbial translocation is through the measurement of serum lipopolysaccharide (LPS). The SMART study revealed that soluble CD14, a marker of monocyte response to LPS, was an independent predictor of mortality in PLWH [12]. This was corroborated by another case-control study by Hunt et al., which concluded that gut epithelial barrier dysfunction independently predicts mortality in individuals with treated HIV infection who also have a history of AIDS [13].

**Figure 1:**
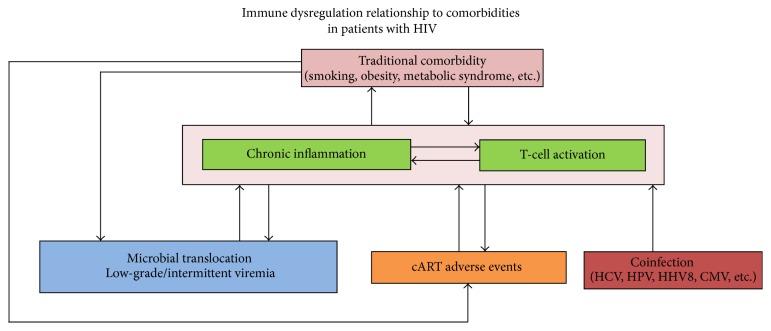
Microbial translocation, low-grade viremia, coinfection, and adverse events from cART all contribute towards chronic inflammation and T-cell activation. This immune dysfunction augments the severity of traditional comorbid conditions. Furthermore, the comorbidities themselves intensify all of the aforementioned factors causing a positive feedback cycle.

Another factor relating to inflammation in PLWH is T-cell function. It is unclear if immune dysregulation leads to inflammation or vice versa. Nonetheless, they are usually present together and both contribute to the burden of comorbid illnesses [14]. Hunt et al. studied the relationship of immune activation and increased CD4 count when HIV was suppressed with cART and found that increased T-cell activation was associated with shorter duration of viral suppression, HCV coinfection, frequent low-level viremia, lower nadir CD4^+^ T-cell counts, and a lower gain in CD4^+^ T-cells [15]. In an elegant study of impaired gut junctional complexes by Tincati et al., a relationship was established between gut damage, HIV viral reservoir, and CD4^+^ response to cART [5]. They concluded that the more damage to the gut and the larger the reservoir, the less the increase in CD4^+^ cells while on suppressive cART [5].  Figure 1 illustrates the factors involved in the chronic inflammatory state and immune dysfunction in PLWH with controlled viral load.

It is also postulated that coinfection is a key contributor to immune dysregulation present in PLWH receiving suppressive cART. This association was well established by Masiá et al., who prospectively studied multiple blood biomarkers of inflammation in monoinfected HIV patients compared to those coinfected with HHV8 [16]. Both groups had a suppressed HIV VL, but inflammation and immune activation were significantly higher in those with HHV8 coinfection [16]. Since HCV is another prevalent virus found in 20–25% of PLWH, it is relevant to study its impact on immune recovery as well. Zaegel-Faucher et al. retrospectively reviewed this data in patients with undetectable VL for at least 3 years and concluded that CD4^+^ percentage and CD4/CD8 ratio were lower in patients coinfected with HCV compared to those with monoinfection, even though they had similar cART regimen and CD4^+^ and CD8^+^ counts at first undetectable HIV VL [6]. Furthermore, Modjarrad and Vermund reviewed the literature up to April 2010 and found that treatment of* Mycobacterium tuberculosis*, syphilis, and other infections significantly decreased HIV VL, even when no cART was used [17]. In another prospective study using CMV PCR, Deayton et al. established a direct correlation of positive PCR findings with new AIDS-defining disorders and mortality in PLWH in the highly active cART era [18]. Lastly, in their review of CMV in PLWH, Barrett et al. summarized the evidence that CMV could be an important cofactor in the development of age-related morbidities in HIV infection [19].

The pivotal SMART trial provided strong evidence of the association of inflammatory biomarkers and coagulation with increased risk of all-cause mortality [20]. The study also showed that interleukin-6 (IL-6) and D-dimer were significantly associated with increased risk of CVD and other causes of death, even in patients on cART [21]. Tenorio et al. conducted a case-control study that concurred with these findings, concluding that soluble inflammatory markers correlated with non-AIDS-defining events in patients virally suppressed on therapy [22].

### 3.2. The Role of Traditional Comorbidity and Coinfection

The new challenges facing the HIV-infected population are non-AIDS-related conditions. A prospective HIV cohort study by the D:A:D group found that the most common comorbidities leading to death in HIV patients were non-AIDS cancers, cardiovascular disease (CVD), and liver disease [23]. Morlat et al. expressed these same findings in a study in France in 2010 [24]. A Swiss HIV cohort study by Hasse et al. revealed that stroke, myocardial infarction, diabetes mellitus (DM), fragility bone fractures, and non-AIDS-defining malignancies were significantly elevated for persons aged ≥65 years [25]. Guaraldi et al. performed a case-control study on cART-experienced patients treated at Modena University, Italy, from 2002 to 2009 [26]. They were compared with age-, sex-, and race-matched adults from the general population [26]. They specifically looked at noninfectious comorbidities (NICMs), which included CVD, hypertension, diabetes mellitus (DM), bone fractures, and renal failure [26]. The study defined polypathology (Pp) as the concurrent presence of ≥2 NICMs and concluded that the prevalence of Pp among HIV patients aged 41–50 years was similar to that among controls aged 51–60 years [26]. Logistic regression models showed that independent predictors of Pp in the overall cohort were age (odds ratio OR, 1.11), male sex (OR, 1.77), nadir CD4^+^ count < 200 cells/*μ*L (OR, 4.46), and cART exposure (OR, 1.01) [26].

Multiple studies have identified HIV as an independent risk factor for acute myocardial infarction (AMI). Freiberg et al. reviewed data from participants in the Veterans Aging Cohort Study that included both HIV-infected and noninfected individuals [27]. This study concluded that infection with HIV was associated with a 50% increased risk of AMI beyond which was explained by recognized risk factors [27]. In two cohorts from the Partners HealthCare System in Boston, Triant et al. compared the rate of AMI in HIV-positive and HIV-negative patients while adjusting for age, gender, race, hypertension, DM, and dyslipidemia [28]. They, too, concluded that there was an increased risk of AMI in patients with HIV, especially in HIV-positive women [28]. Lastly, Okeke et al. reviewed the hospital discharge data from the Nationwide Inpatient Sample from 2002 to 2012 looking specifically for patients with AMI or stroke [29]. They used multivariable logistic regression to evaluate the association between HIV and in-hospital death [29]. They found that patients with a history of AIDS were significantly more likely to die in-hospital after AMI and stroke than noninfected patients [29]. This disparity was not observed when infected patients without a history of AIDS were compared to noninfected patients [29].

Malignancies that are considered AIDS-related such as Kaposi's sarcoma, primary central nervous system lymphoma, and cervical cancer have dramatically declined since the advent of suppressive ART [30]. Furthermore, the incidence of non-AIDS-related malignancies (NARM) including anal cancer, hepatocellular carcinoma, head and neck cancers, lung cancers, non-Hodgkin's lymphoma, and melanoma has increased so significantly that they now represent one of the most common causes of death in PLWH in the USA [31]. Immune dysregulation and chronic inflammation in PLWH can promote increased cell proliferation and generate potentially damaging reactive oxygen species [32]. The immune dysfunction associated with HIV infection may also lead to impaired immune surveillance with an impaired ability to both detect and eliminate early tumor cells [33]. Powles et al. reported from their large prospective cohort that a nadir CD4^+^ count < 200/microL had a significant association with NARM [34]. Certain traditional risk factors for NARM are more prevalent in PLWH [35, 36]. Some of these include smoking, with its subsequent increase in lung cancer, as well as hepatitis B and C viruses, with their associated risk for hepatocellular carcinoma [35, 36]. There is a higher incidence of Hodgkin's lymphoma in PLWH than the general population, and it is often associated with EBV coinfection [37]. Probably the highest increase in cancer types in HIV-infected patients compared to those that are noninfected is related to HPV [38]. Associated cancer types with this coinfection include anal cancer, head and neck cancer, and cervical cancer (which is an AIDS-related malignancy) [38].

The incidence of DM is also increased in PLWH [39]. Inflammation and HIV lipodystrophy involve adipose tissue redistribution, mitochondrial dysfunction, altered differentiation of adipocytes, and increased adipocyte lipolysis [40, 41]. This leads to altered adipokine secretion, as well as the release of proinflammatory cytokines and free fatty acids [40, 41]. This, in turn, exacerbates chronic inflammation, dyslipidemia, and insulin resistance [40, 41].

The effect of HIV on liver disease was well characterized by Towner et al. in their case-control study which concluded that HIV-infected individuals have a higher risk of hepatic dysfunction and hepatic-related death compared to those without HIV infection, even with adjustment for known hepatic risk factors [42]. Marchetti et al. revealed that HIV coinfected patients, mainly HCV, with higher TNF-*α* plasma levels had a 13-fold increase in the risk of progression to a Fib-4 > 1.45 [43]. However, these patients were not receiving ART [43]. A more recent study suggests that ART with good immune reconstitution can slow down liver fibrosis in HIV/HCV coinfected patients [44].

### 3.3. Chronic Effects of cART

With the ever-emerging population of patients with long-term controlled HIV infection, the chronic effects of cART are becoming better understood. It has already been well established that a high proportion of patients who delay cART until the CD4^+^ count drops below 200 cells/mm^3^ do not achieve a normal CD4^+^ count, even after a decade of effective therapy [45]. In fact, the DHHS guidelines now recommend usage of cART in all patients regardless of CD4^+^ count [46]. Furthermore, studies have suggested that CD4^+^ cell recovery from cART is both slower and less pronounced in elderly patients, which provides further credence to the concept that early cART initiation will lead to the best possible degree of immune regulation [47, 48]. These guidelines are supported by multiple randomized controlled studies like START and TEMPRANO proving that the earlier ART is started the better the immune system is preserved and the less inflammation is present, with subsequently less mortality and morbidity [49, 50].

cART must also be considered a potential cause of adverse events and, therefore, a possible contributor towards inflammation and aging in those with controlled HIV infection [51]. Extra attention to drug toxicity from cART must be given to the elderly HIV patient due to the high degree of concurrent medication use and, thus, the greater potential for harmful drug-drug interactions, as well as age-related changes in renal and hepatic function that could affect drug clearance.

Certain regimens have been linked to toxicities that increase the risk for comorbidities. This paper has already mentioned the role of comorbidity in persistent inflammation. cART can further negatively contribute towards these processes by augmenting the severity and effects of comorbid conditions. Friis-Møller et al. studied the association between cART and CVD risk factors and found that nonnucleoside reverse transcriptase inhibitor (NNRTI) and protease inhibitor (PI) use was linked to elevated total cholesterol [52]. The D:A:D Study Group added further to this subject by investigating the association between certain cART drugs and acute myocardial infarction (AMI) [53]. The study revealed that abacavir, didanosine, indinavir, and lopinavir-ritonavir carried the most significant association [53]. Lang et al. further contributed to this subject, stating that cumulative exposure to PIs, especially (fos)amprenavir with or without ritonavir and lopinavir with ritonavir, had increased risk for AMI [54]. However, saquinavir was found not to have this association [54]. Lopinavir and ritonavir were also shown to increase the risk for AMI in a study conducted by Durand et al. [55].

Abacavir increasing the risk for AMI has been a topic of debate. A paper by Marcus et al. addressed this issue and concluded that abacavir increases the risk of CVD by 2.2 times [56]. Two other studies, one by the D:A:D group and another by Obel et al., also confirmed that abacavir is associated with AMI [57, 58]. In their cohort study, Choi et al. concluded that recent abacavir exposure increased the risk for cardiovascular events and that tenofovir (TDF) was associated with heart failure [59]. Costagliola et al., however, reviewed the literature on abacavir and CVD and reported that due to confounding variables and selection bias, it is impossible to neither verify nor repudiate a correlation between the two [60]. Many studies claim that abacavir use carries no independent risk for AMI [61–64]. Bedimo et al. found no association between MI use and abacavir use but did report that abacavir use was more common than TDF use in patients with chronic kidney disease, which itself is an independent risk factor for CVD [62]. Nonetheless, the DHHS guidelines suggest avoiding abacavir and lopinavir/ritonavir in patients at high risk for cardiovascular events [46].

Other associations between certain cART toxicities and comorbidities have been established in the literature. Ryom et al. found that cumulative use of stavudine, didanosine, (fos)amprenavir, and TDF was independently associated with higher rates of end stage liver disease and hepatocellular carcinoma [65]. In their prospective observational study, De Wit et al. showed that stavudine and zidovudine were linked with insulin resistance, perhaps due to toxic effects on the mitochondria [66]. Moreover, TDF and efavirenz have been linked with decreased bone marrow density [67]. In their meta-analysis, Brown and Qaqish concluded that PI-treated patients had increased odds of osteoporosis than non-PI-treated patients [68]. Also, a study by Perrot et al. linked TDF use to osteomalacia from proximal renal tubular dysfunction [69]. Herlitz et al. further studied the potential adverse events of TDF use by documenting reversible acute tubular necrosis and mitochondrial dysmorphic changes in 13 cases of TDF-associated nephrotoxicity that were on therapy for a mean of 19.6 months [70]. Two controlled, double-blind phase 3 studies by Sax et al. compared the adverse events associated with TDF with those of its newer formulation tenofovir alafenamide (TAF) [71]. They concluded that TAF has less renal and bone adverse events than TDF because this tenofovir prodrug causes a 90% decrease in tenofovir plasma concentration [71]. Also, TAF was noninferior to TDF in suppressing HIV VL [71]. Additionally, Leeansyah et al. studied telomerase activity and length in vitro by looking at peripheral blood mononuclear cells (PBMCs) from HIV-infected patients receiving a NRTI-containing regimen and found that they had significantly lower telomerase activity than both HIV-uninfected patients and HIV-infected patients receiving a non-NRTI-containing regimen [72]. Telomerase length was inversely associated with age, as well as the total duration of NRTI-containing therapy [72]. This study concluded that NRTIs at therapeutic concentrations, specifically TDF, inhibit telomerase activity and this leads to its accelerated shortening in activated PBMCs, which could play a role in the enhanced aging of PLWH [72]. This is important because some studies have reported that the HIV virus itself causes shortening of leukocyte telomere length, and, therefore, TDF use may further contribute towards this same process [73, 74].

### 3.4. Managing Inflammation in the HIV Patient

The management of cART in the HIV-infected patient must focus on the need not only to suppress the serum HIV VL but also to target potential viral reservoirs in order to decrease immune activation and the chronic inflammatory response. One of these possible reservoir sites is within the central nervous system (CNS). In their CROI abstract, Anderson et al. examined the effect of cART therapy with good CNS penetration in virally suppressed patients by documenting inflammatory markers in cerebrospinal fluid (CSF) [75]. They found that, during suppressive cART, regimens that are estimated to have better distribution into the CNS were associated with decreased levels of CXCL10 and TNF-*α* within the CSF and, therefore, less inflammation [75]. IL-6 remained elevated even in regimens with good CNS penetration [75].

Another potential site for these reservoirs is within the gut mucosa [76–78]. Gandhi et al. studied the effects of adding another antiretroviral agent to an already suppressive regimen [79]. They measured any changes in the level of residual viremia in patients with VL < 20 copies/mL after adding raltegravir (RAL) for 12 weeks [79]. They found that intensification with RAL did not reduce the amount of residual viremia [79]. Although a study by Yukl et al. echoed the same results for a RAL-containing intensification on the VL in plasma, it did reveal a decrease in T-cell activation within the gut [77]. Perhaps this is because RAL concentrations remain significantly higher in the gut-associated lymphoid tissue (GALT) and gastrointestinal tract than in the plasma, causing less viral replication at these sites [78]. Hatano et al. concurred with the observation that RAL intensification decreases low-level viral replication, as evidenced by their measurement of an increase in the level of 2-long terminal repeat (2-LTR) circles in these patients [80]. Although this is a promising treatment option, other accounts claim that intensification with RAL did not increase overall CD4^+^ count nor lower HIV proviral DNA in gut CD4^+^ cells [76].

Other studies have investigated changes in inflammatory markers after switching suppressive cART to RAL [81–84]. The SPIRAL trial took controlled patients on a boosted protease inhibitor (PI) and then randomly switched them in a 1 : 1 fashion to RAL [82]. Results at 48 weeks after the randomized switch revealed significant changes in several cardiovascular biomarkers that could not be completely explained by lipid changes [82]. Lake et al. reported a decrease in sCD14 in obese women who were changed to RAL who previously had controlled VL on a PI or NNRTI [84]. A study by Silva et al. found a similar decrease in inflammatory markers in virologically suppressed patients that were switched to RAL from an enfuvirtide-based cART [83]. Lastly, Gupta et al. changed patients virally suppressed on efavirenz to RAL and documented that the switch group had decreased C-reactive protein, sCD14, and renal function and increased levels of sCD163 [85]. This suggests that, compared to the continuation group, RAL may more positively impact monocyte activation, but it is not superior in its effects on endothelial function. Furthermore, RAL could contribute more towards nephrotoxicity than efavirenz [85].

Intensification with maraviroc (MVC) has also been tested in virally suppressed patients to determine its effects on CD4^+^ cell restoration and immune regulation. Cuzin et al. added MVC for 24 weeks to the cART of 60 patients with CD4^+^ < 350 cells/mm [86]. They found that these patients were able to achieve an increase in CD4^+^ slopes [86]. Hunt et al. also studied MVC intensification in patients with VL < 48 copies/mL and CD4^+^ < 350 cells/mm [87]. Compared with the placebo group, MVC-treated subjects had increased peripheral blood CD8^+^ cells, sCD14, and neutrophils, with a lesser effect in suppressing CD4^+^ cell activation by week 24 [87]. Lastly, Belaunzarán-Zamudio et al. compared the immune recovery in patients with CD4^+^ < 100 cells/mm who received either MVC intensification or placebo to a standard cART regimen [88]. Those on MVC retained higher rates of CCR5^+^, CD4^+^, and CD8^+^ cells and had no effect on IRIS occurrence [88].

There have been a small variety of head-to-head trials in treatment naïve patients in order to assess which regimens are most efficacious in diminishing immune activation. Hileman et al. examined markers of inflammation and monocyte activation in a randomized controlled blinded study of single tablet regimen of cobicistat/elvitegravir/emtricitabine/TDF versus efavirenz/emtricitabine/TDF [89]. They concluded that the elvitegravir-containing regimen had a greater decrease in sCD14, hsCRP, and Lp-PLA2 levels compared to the efavirenz-containing regimen [89]. The effects on the inflammatory response by MVC versus TDF were examined in an abstract submitted by Chan et al. [90]. They found that initiating cART with MVC caused a greater increase in CD4^+^ cells, a smaller decrease in CD8^+^ cells, and a smaller increase in CD4^+^/CD8^+^ ratio compared to TDF [90]. However, there was no difference in changes in inflammatory biomarkers between the two regimens [90]. Papakonstantinou et al. compared the inflammatory effects on treatment naïve patients assigned a regimen of either TDF/emtricitabine/efavirenz or abacavir/lamivudine/efavirenz [91]. The results of the study revealed that the TDF-containing regimen caused a decrease in platelet-activating factor (PAF) levels and lipoprotein-associated phospholipase A2 (Lp-PLA2) activity [91]. The abacavir-containing regimen showed no change in baseline PAF and an increase in Lp-PLA2, which may be the reasoning behind abacavir-associated cardiovascular adverse events [91]. Lastly, Barrios et al. studied TDF + didanosine in patients who were naïve to treatment or who simplified a prior suppressive cART regimen [92]. They established that although VL was undetectable, a decline in CD4^+^ count was evident, which they theorized could be due to an imbalance in adenosine metabolites within CD4^+^ cells [92]. A summary of the studies regarding cART intervention to manage the chronic inflammatory state in HIV patients can be found in Table 1.

There have also been promising studies suggesting alternate therapies to treat chronic inflammation. Multiple published trials revealed that rosuvastatin showed benefit in reducing inflammation markers and immune activation [93–95]. Wooten at al. examined the effect of healthy diet and exercise on inflammation in HIV patients with undetectable VL and dyslipidemia [96]. They found that these interventions effectively reduced plasma Lp-PLA2 mass [96]. Furthermore, patients should be counseled to stop smoking. Valiathan et al. compared HIV-infected smokers and nonsmokers that had documented viral suppression on cART to HIV-uninfected smokers and nonsmokers [97]. They found that smoking and HIV infection both independently influence T-cell immune activation and function, and together they present the worst immune profile [97]. Villar-García et al. conducted a double-blind, randomized, placebo-controlled trial of* Saccharomyces boulardii* in 44 patients with viral load of <20 copies per milliliter for at least 2 years. They found that this fungus was very effective at decreasing microbial translocation and inflammation parameters [98]. Another innovative approach at non-cART intervention was a 12-week, single-arm, open-label study, whereby Sereti et al. tested the efficacy of IL-7 adjunctive therapy on T-cell reconstitution in peripheral blood and gut mucosa in 23 cART suppressed HIV-infected patients with incomplete CD4^+^ recovery [99]. They observed that administration of r-hIL-7 improved the gut mucosal abnormalities of chronic HIV infection and attenuated the systemic inflammatory and coagulation abnormalities associated with the said gut disease [99].

In the DHHS guidelines, the DHHS does not advocate for cART intensification because this method has not shown consistency in reducing the immune activation nor in increasing T-cell recovery [46]. Furthermore, the guidelines do not recommend switching cART regimens in virally suppressed patients due to the lack of substantial evidence of its effects on the chronic inflammatory response [46]. Also, no instructions for the trending of immune markers in those with chronically suppressed HIV infection have been incorporated into the guidelines because there has yet to be any proven predictability value in morbidity and mortality from doing so [46]. Because of this, the DHHS explains that clinicians should aim at monitoring modifiable risk factors for comorbid conditions [46]. Also, it is recommended that patients with poor CD4^+^ recovery should be worked-up for a modifiable cause, most notably adverse events from medications [46].

## 4. Conclusion

Overall, the harmful effects of chronically suppressed HIV infection on inflammation and immune activation must always be considered when managing PLWH. As the population of HIV-infected patients ages, the long-term effects of immune dysregulation will augment the severity of other non-AIDS-related comorbidities. Furthermore, the potential toxic events from certain cART could also contribute towards increased morbidity and mortality compared to the general population in the long run. Although knowledge about this subject is increasing, real life data to suggest appropriate ways to manage this inflammation related to HIV is seriously lacking. There are not enough studies done to establish a consensus to which antiretroviral drug class is best at suppressing the inflammatory response. This is due to the difficulty in finding the right means to accurately measure the chronic inflammatory state. Clinicians will most likely have to settle for a battery of tests that will be validated from collectively trending them over time. At this point, a practical approach to the management of PLWH to best preserve the immune system and control the chronic inflammatory process should be to (1) initiate cART as early as possible, (2) both prevent and treat coinfection, (3) aggressively treat any comorbid condition, (4) advise patients to stop smoking and increase physical activity, and (5) switch cART to the least toxic regimen. It is absolutely essential to conduct more clinical trials to determine which regimens are both most effective and safest at controlling the inflammatory response.

## Figures and Tables

**Table 1:** Antiretroviral interventions that affect immune dysregulation in patients on cART.

Author	Method	Year of publication	Primary endpoint	Number of patients	Conclusion
Barrios et al. [92]	Retrospective cohort study	2005	HIV-infected individuals who initiated a protease inhibitor-sparing regimen were retrospectively assessed by analyzing viral load and CD4^+^ count.	570	Patients receiving ddI + TDF-based combinations showed a decrease in CD4^+^ count despite having an undetectable viral load.

Gandhi et al. [79]	Prospective, randomized, placebo- controlled trial	2010	Groups were randomized with RAL intensification or placebo, and plasma HIV-1 RNA was averaged between weeks 10 and 12.	53	12 weeks of RAL intensification did not demonstrably reduce low-level plasma viremia in patients on currently recommended cART.

Hatano et al. [81]	Prospective, randomized, placebo- controlled trial	2011	Patients received 24 weeks of cART intensification with RAL or placebo. End points were defined as change in the % CD38^+^HLA-DR^+^CD8^+^ T-cells in PBMCs and number of patients with undetectable viral load.	30	RAL intensification did not have a significant effect on immune activation or HIV-specific responses in PBMCs or gut-associated lymphoid tissue.

Chege et al. [76]	Prospective, randomized, placebo- controlled trial	2012	Patients received 48 weeks of cART intensification with RAL or placebo. After week 48, all patients were given RAL until week 96. Blood and sigmoid biopsies were sampled to document CD4^+^ count as well as HIV-1 proviral DNA load.	24	RAL did not cause any significant difference in CD4^+^ count and blood and gut HIV-1 proviral load compared to placebo.

Martínez et al. [82]	Prospective, randomized, open-label study	2012	Changes in fasting lipids, hsCRP, MCP-1, osteoprotegerin, IL-6, IL-10, TNF-*α*, ICAM-1, VCAM-1, E-selectin, P-selectin, adiponectin, insulin, and D-dimer were documented for 48 weeks in patients on a RAL-boosted protease inhibitor and those who were switched to RAL.	273	Significant decreases in cardiovascular biomarkers were reported in patients who were switched to RAL.

Cuzin et al. [86]	Prospective, open-label study	2012	Patients were put on MVC intensification and changes in CD4^+^ slopes were documented.	60	MVC intensification caused enhancement of CD4^+^ cell slopes in patients with history of poor immune restoration.

Silva et al. [83]	Prospective, randomized, open-label study	2013	Plasma IL-6, hsCRP, and D-dimer levels were documented at baseline and at weeks 24 and 48.	164	At week 24, a significant decrease in IL-6 and D-dimer level was seen in the immediate RAL switch arm compared with the deferred switch arm. At week 48, the deferred RAL switch arm had a decrease in all measured biomarkers.

Gupta et al. [85]	Prospective, randomized, placebo- controlled trial	2013	Flow-mediated dilation, 25(OH) vitamin D, PTH levels, total cholesterol, hsCRP, serum ALP, sCD14 levels, and renal function were compared for 24 weeks between patients on EFV and those switched to RAL.	30	The RAL switch group showed a decrease in total cholesterol, hsCRP, serum ALP, sCD14 levels, and renal function.

Hunt et al. [87]	Prospective, randomized, placebo- controlled trial	2013	Patients with MVC intensification were compared to a placebo group by measuring % CD38^+^HLA-DR^+^, CD8^+^, CD4^+^, CCR5 ligand levels, plasma lipopolysaccharide, sCD14 levels, and neutrophils.	45	During MVC intensification, plasma lipopolysaccharide declined and sCD14 and neutrophils increased in blood and rectal tissue.

Hatano et al. [80]	Prospective, randomized, placebo- controlled trial	2013	Patients received 24 weeks of cART intensification with RAL or placebo. 2-LTR circles by droplet digital polymerase chain reaction were documented at weeks 0, 1, 2, and 8.	31	RAL intensification resulted in a rapid increase in the level of 2-LTR circles in a proportion of subjects, indicating that low-level viral replication persists in some individuals even after long-term cART.

Lake et al. [84]	Prospective, randomized open-label study	2014	Changes in sCD14 and other inflammatory biomarkers in virologically suppressed HIV-infected women were documented for 48 weeks.	37	A switch to RAL from a protease inhibitor or nonnucleoside reverse transcriptase inhibitor was associated with a statistically significant decline in sCD14.

Papakonstantinou et al. [91]	Prospective, open-label study	2014	Treatment naïve patients were assigned a regimen of TDF/FTC/EFV or ABC/3TC/EFV. Inflammatory markers, metabolic enzymes, and HIV-implicated cytokines were collected and compared for a 12-month period.	18	The TDF-containing regimen caused a decrease in PAF levels and Lp-PLA2. The ABC-containing regimen caused increased Lp-PLA2.

Hileman et al. [89]	Prospective, randomized, double-blinded study	2015	sCD14, sCD163, sTNF-RI, IL-6, hsCRP, and Lp-PLA2 were compared over 24 and 48 weeks between patients on EVG and EFV.	200	EVG seems to have better effects on immune activation than EFV.

Belaunzarán-Zamudio et al. [88]	Prospective, randomized, placebo- controlled trial	2016	Flow cytometry was used to characterize the maturation phenotype, CCR5 ligand level expression, and T-cell activation at weeks 0, 4, 12, 24, and 48 in patients who received MVC intensification. CD4^+^ and CD8^+^ cell reactivity was also determined by intracellular expression of IFN-*γ*, TNF-*α*, and CD40 ligand at weeks 0, 4, and 12.	40	Those on MVC intensification retained CD4^+^ and CD8^+^ cells. Treatment had no effect on the occurrence of IRIS.

Chan et al. [90]	Prospective, randomized, double-blinded study	2016	32 biomarkers and bone mineral density of the hip were measured at weeks 0 and 48 for treatment naïve patients on MVC or TDF.	230	Initiating cART with MVC compared to TDF caused a greater increase in CD4^+^ count and smaller decline in CD8^+^ count, but less rise in CD4^+^/CD8^+^ ratio. There was no difference in inflammatory biomarkers.

ddI: didanosine; TDF: tenofovir; RAL: raltegravir; HLA-DR: human leukocyte antigen-antigen D related; PBMCs: peripheral blood mononuclear cells; cART: combination antiretroviral therapy; hsCRP: high-sensitivity C-reactive protein; MCP-1: monocyte chemoattractant protein 1; IL-6: interleukin-6; IL-10: interleukin-10; TNF-*α*: tumor necrosis factor-alpha; ICAM-1: intercellular adhesion molecule-1; VCAM-1: vascular cell adhesion molecule-1; MVC: maraviroc; PTH: parathyroid hormone; ALP: alkaline phosphatase; EFV: efavirenz; CCR5: C-C chemokine receptor type 5; 2-LTR: 2-long terminal repeat; Lp-PLA2: lipoprotein-associated phospholipase A2; FTC: emtricitabine; ABC: abacavir; 3TC: lamivudine; PAF: platelet-activating factor; sTNF-RI: soluble tumor necrosis factor *α* receptor I; EVG: elvitegravir; IFN-*γ*: interferon-gamma; IRIS: immune reconstitution inflammatory syndrome.
